# Osteogenic Differentiation of adipose mesenchymal stem cells with BMP-2 embedded microspheres in a rotating bed bioreactor

**DOI:** 10.1186/1753-6561-5-S1-P74

**Published:** 2011-11-22

**Authors:** Stefanie Boehm, Yael Lupu, Marcelle Machluf, Cornelia Kasper

**Affiliations:** 1 Institute of Technical Chemistry, Leibniz University of Hannover, Callinstr. 5, 30167 Hannover, Germany; 2 Faculty of Biotechnology and Food Engineering, Technion, Haifa, Israel

## Introduction

Bone tissue engineering aims at the generation of functional bone tissue for replacement of defect bone tissue in order to reestablish normal function. For this purpose mesenchymal stem cells (MSCs) are widely used since they can be isolated from different sources and can easily be differentiated *in vitro* into the mesenchymal lineages [1,2].

The aim of this work was to study the effect of growth factor BMP-2 on the osteogenic differentiation of human mesenchymal stem cells from adipose tissue. The cells were cultivated in a rotating bed bioreactor system (Z^®^RPD system, Zellwerk GmbH) on the aluminium oxide based ceramic Sponceram^®^ for four weeks. The ceramic discs were loaded with PLGA microspheres releasing BMP-2. For this experiments a system was used which allows the parallel cultivation of four bioreactors. The used bioreactor containers were manufactured of disposable material for single use application. During the cultivation glucose and lactate concentrations were measured and after the experiments histological stainings were performed (DAPI, von Kossa and alizarine red). Furthermore the concentration of alkaline phosphatase was measured in medium samples and mRNA of the cells was isolated to perform RT-PCR to investigate the expression of different bone markers.

## Materials and methods

### Biomaterial

The Sponceram^®^ biomaterial consists of AlO_2_. It has a macroporous structure (600 µm) with a microporous surface and a porosity of 85 %. For the cultivations scaffolds with a diameter of 65 mm and a thickness of 3 mm were used. The PLGA microspheres were produced by solvent evaporation and at a size of about 50 µm. The BMP-2 concentration in the microspheres was about 0.05 µg per mg PLGA. The total amount of BMP-2 in the dynamic cultivations was 2.5 µg in each bioreactor. The BMP-2 release and the concentration in the medium was measured with a BMP-2 ELISA (Quantikine, R&D Systems) The BMP-2 concentration in medium samples of bioreactor 4 decreases almost linear from 875 pg/ml to zero pg/ml in the first 16 days of the dynamic cultivation.

### Cells

The adipose tissue derived mesenchymal stem cells (adMSC) were isolated by enzymatic treatment under GMP conform conditions in a laboratory of the Red Cross in Linz, Austria. For the dynamic cultivation the isolated cells were expanded in cell factories (Nunclon™ Δ Cell Factory) and the dynamic cultivations were performed with cells in passage 5. For the cultivations 5x10^6^ cells were used in every bioreactor.

### Bioreactor

The Z^®^RPD bioreactor system consists of four disposable rotating bed bioreactors and is working in a perfusion mode. The reactor is equipped with a Sponceram^®^ ceramic, which is served as a rotating bed. In the standard operation mode the bioreactor is half filled with media (50 mL) and the Sponceram^®^ is rotating with 0.5 rpm. This way the cells on the ceramics have alternating contact to medium and gas-filled headspace improving cell nutrition and oxygen supply. The aim of the parallel cultivation of four bioreactors was, to test the reproducibility of the system, which is important for GMP standards in clinical application. The bioreactors were integrated in a GMP conform breeder with a control unit for GMP conform documentation and evaluation of pH, oxygen and temperature. Furthermore a sterile sampling of media during the cultivation is possible. The dynamic cultivations were performed over a time period of 28 days and the cells were cultivated in osteogenic medium with dexamethasone, l-ascorbate and β-glycerol phosphate.

## Results

During 28 days of dynamic cultivation about 200 mg glucose were consumed in each bioreactor. The glucose consumption increased during the cultivation indicating a continuous cell growth on the ceramics. Also DAPI staining of the cell seeded ceramics showed a high density of cells on the scaffolds. Furthermore the concentration of alkaline phosphatase was measured daily (Sigma *fast*^TM^, Sigma Aldrich). The amount of the secreted alkaline phosphatase decreased during the cultivation period which is an indication for advanced osteogenic differentiation. Also the expression of various genes involved in the osteogenic differentiation of cells was verified. Different intensities of the gene expression in the four bioreactors was observed; especially the gene expression in bioreactor 1 was obviously higher. SEM pictures of the cell seeded ceramics showed thick cell layers on Sponceram^®^. Small round beads and a fibrous structure were noticeable. The beads might be cells, which are embedded in the extracellular matrix and the fibrous structure could be a hint for Collagen reposition. To verify the osteogenic differentiation of the cells von Kossa- and alizarine red stainings were performed. Both stainings showed high amounts of calcified matrix on all four ceramics. Also the alkaline phosphatase staining (Sigma *fast* BCIP, Sigma Aldrich) was successful and showed high amounts of secreted alkaline phosphatase on all four scaffolds.

## Conclusion

The glucose consumption increased during the cultivation indicating a continuous cell growth and the consumption was almost the same in all bioreactors. Also the DAPI staining of the ceramics showed a homogenous spreading of the cells on the Sponceram^®^ ceramics.But only in two bioreactors a typical alkaline phosphatase concentration curve could be verified in collected medium samples. The histological staining showed matrix calcification of the cells on every ceramic. Also the expression of typical bone markers was shown. In summary, osteogenic differentiation of adMSC on Sponceram^®^, caused by BMP-2 released from PLGA microspheres, was demonstrated and the reproducibility of the osteogenic differentiation in four parallel cultivations was demonstrated.

**Figure 1:**
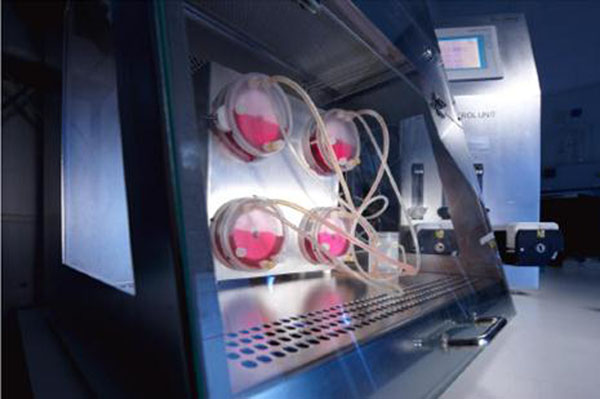
ZRP® bioreactor system (Zellwerk GmbH) with the special setup for performing four parallel cultivations.
